# Supplementary figures and images for: Spop deficiency impairs adipogenesis and promotes thermogenic capacity in mice
Source: PLoS Genet. 2024 Dec 16;20(12):e1011514. doi: 10.1371/journal.pgen.1011514 (PMC11684654; doi:10.1371/journal.pgen.1011514)

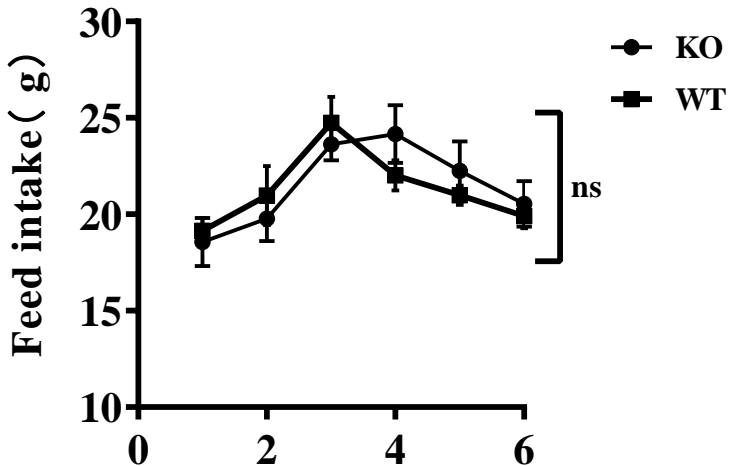

Supplement: S1 Fig — (PDF) [file pgen.1011514.s001.pdf]

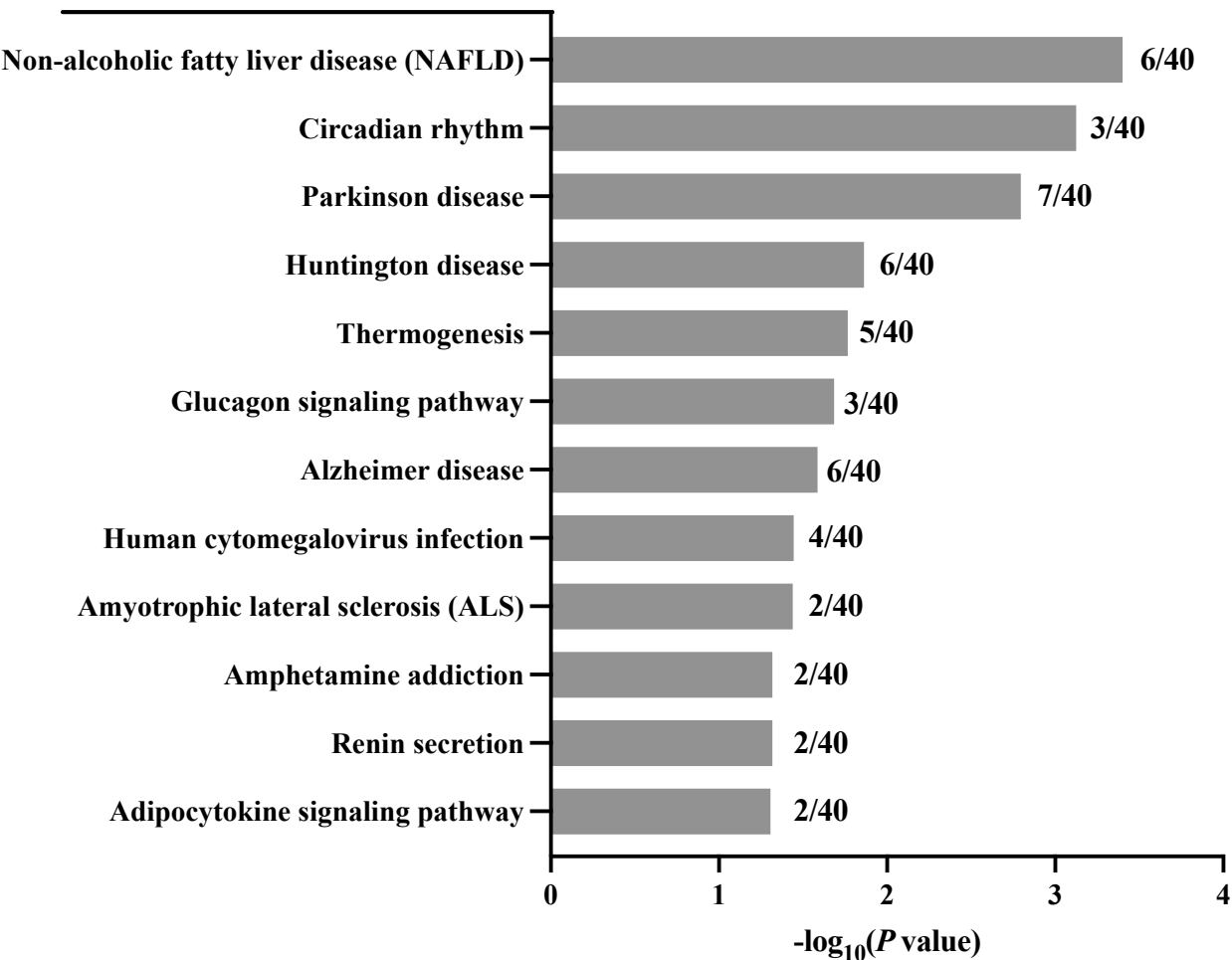

Supplement: S2 Fig — (PDF) [file pgen.1011514.s002.pdf]

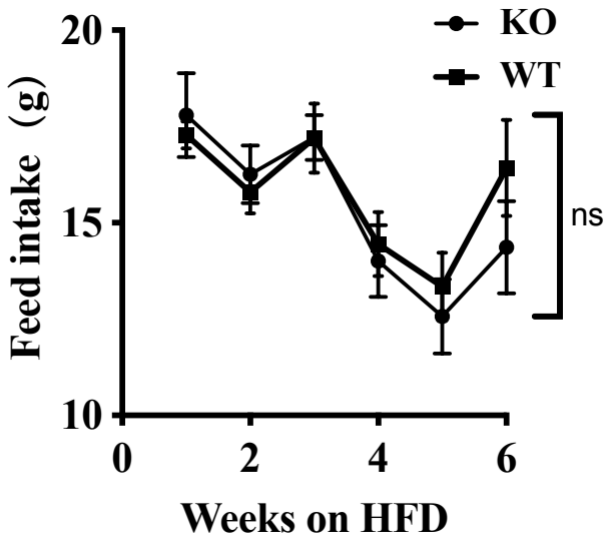

Supplement: S3 Fig — (PDF) [file pgen.1011514.s003.pdf]

**a**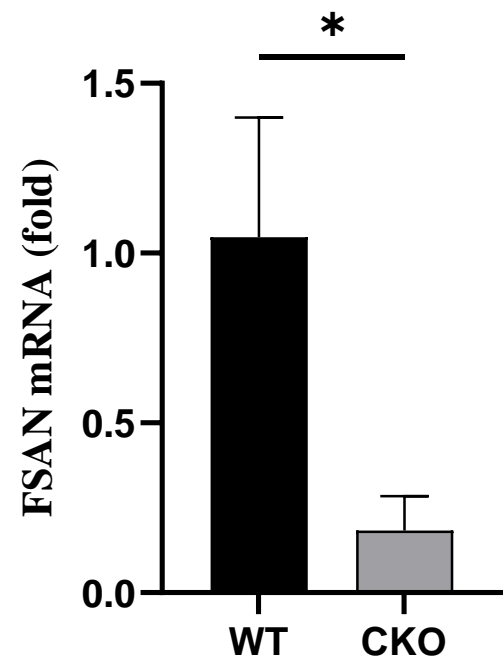**b**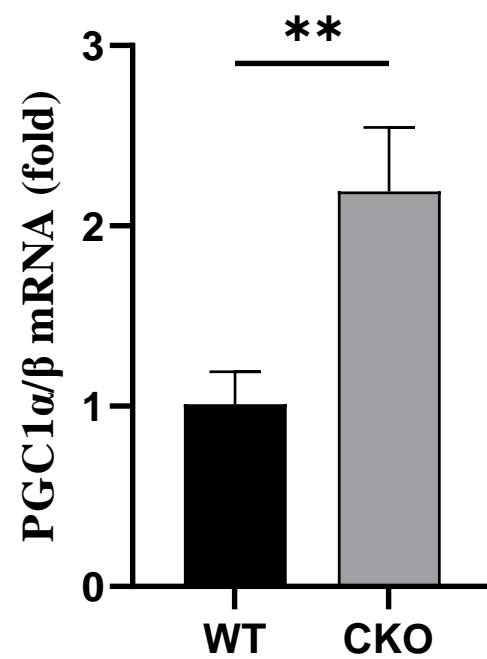

Supplement: S4 Fig — (PDF) [file pgen.1011514.s004.pdf]

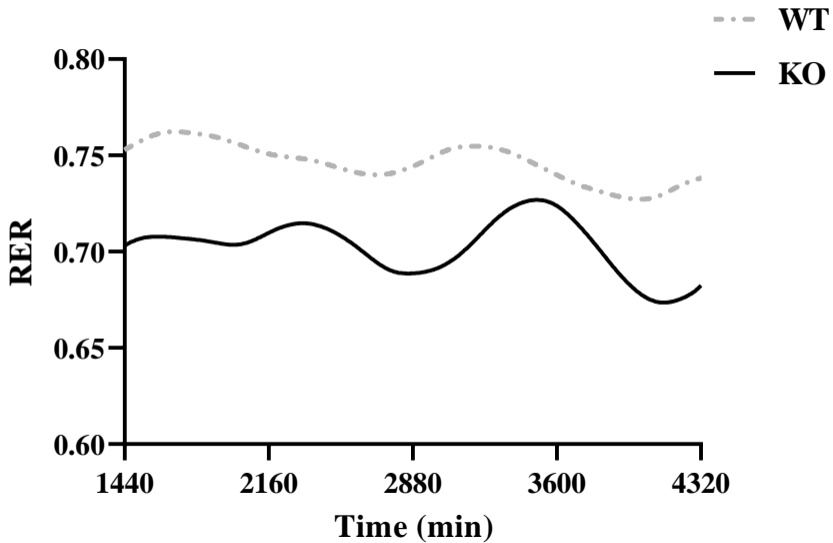

Supplement: S5 Fig — (PDF) [file pgen.1011514.s005.pdf]
