# Supplementary material for: Spop deficiency impairs adipogenesis and promotes thermogenic capacity in mice
Source: PLoS Genet. 2024 Dec 16;20(12):e1011514. doi: 10.1371/journal.pgen.1011514 (PMC11684654; doi:10.1371/journal.pgen.1011514)
Supplement: Attachment — Submitted filename: Response to Reviewers.docx [file pgen.1011514.s009.docx]

Reviewer's Responses to Questions

**Comments to the Authors:
Please note here if the review is uploaded as an attachment.**

Reviewer #1: As the adaptor protein that determines substrate specificity of the Cul3-SPOP-Rbx1 E3 ligase complex, SPOP is involved in numerous biological processes. However, its physiological connections with adipogenesis and thermogenesis remain poorly understood. In the current study, we report that conditional knockout of Spop in mouse results in substantial changes in protein expression, including the upregulation of a critical factor associated with thermogenesis, UCP1. Loss of SPOP also led to defects in body weight gain. In addition, conditional knockout mice exhibited resistance to high-fat-diet-induced obesity. Proteomics analysis found that proteins upregulated in the knockout mice are primarily enriched for functions in glycolysis/gluconeogenesis, oxidative phosphorylation, and thermogenesis. Furthermore, Spop knockout mice were more resilient in cold tolerance assay compared with the wild-type controls. Finally, knockdown of SPOP efficiently impaired adipogenesis in NIH3T3-L1 cells and the expression of associated genes. Collectively, these findings demonstrate critical roles of SPOP in regulating adipogenesis and thermogenic capacity in mouse. There are a number of issues for this work.

Dear Editors

Thank you very much for your email of 3rd Sep regarding the responses of the reviewers to our manuscript " *Spop* Deficiency Impairs Adipogenesis and Promotes Thermogenic Capacity in Mice" (PGENETICS-D-24-00780).

We appreciated all the responses and also thank the reviewers for their insightful comments concerning our work. We have considered their comments carefully and have answered all the points they raised. In addition, we have modified the author list to reflect the latest contributions made to the manuscript during revision.

For the data underlying the figures and results presented in the manuscript, we are currently working on it to submit it to NCBI. We do not need to ask for an exemption for the data availability. There is something wrong with the data format so we have not submitted them to NCBI successfully yet. I think we will figure out this problem and get the accession numbers in ten days. We promise that all the data will be available via a public repository according to the PLOS Genetics data availability policy and add the accession numbers to the proof if this paper is accepted.

All the best

Qinghe Li, for the authors

1. The manuscript heavily focuses on well-known pathways and mechanisms related to thermogenesis and adipogenesis without providing significant novel insights.

Response: We thank this referee for valuable suggestions to improve our manuscript. The novelty of the current study relies on the new function of SPOP in the regulation of adipogenesis and thermogenic capacity. As the adaptor protein which determined the substrate specificity of the Cul3-SPOP-Rbx1 E3 ligase complex, SPOP is widely involved in various biological processes. The novel determinants of adipogenesis and thermogenic capacity by SPOP discovered here significantly expand upon previous studies and provide mechanistic insights into how SPOP is involved in the regulation of various biological processes.
2. Metabolic cage study should be included. The study primarily uses a conditional knockout model and in vitro cell lines. While these models are valuable, the lack of additional validation in other models or human tissues limits the generalizability of the findings.

Response: We thank the reviewer for the helpful suggestions to improve our manuscript.

We have included the data from the metabolic cage study in Figure 4. In the revision, we also added the infrared thermal images of both WT and *Spop* CKO mice. Our results showed that the *Spop* CKO mice are more resistant to the cold environment and they exhibit higher surface temperatures compared to the wild-type mice (Fig 4b).

For the effect of depletion of SPOP on adipocyte differentiation, we present the data in primary adipocyte instead of the data in NIH3T3-L1 cells in the revision. The same as what we have observed in NIH3T3-L1 cells, the depletion of SPOP inhibited preadipocyte differentiation (Fig 5).

In the current study, we clearly showed the regulation of adipogenesis and thermogenic capacity by SPOP deficiency using mice as a model. We respectfully argue that the scientific question we focused on in this study could be efficiently explained by the above findings presented in this manuscript. We hope that more and more work will be carried out, including in humans and other model animals, to further explore the role of SPOP in the regulation of adipogenesis and thermogenesis. We have added the relevant information to the discussion.
3. The manuscript lacks detailed information on the statistical methods used for data analysis.

Response: We thank the reviewer for pointing out this. We have added the following method of statistical analysis into “Materials and Methods”.

Statistical comparisons between two groups were made using the two-tailed Student’s t test; comparisons between three or more groups were made by two-way ANOVA. All results are presented as the mean ± s.e.m. In all analyses, *p* < 0.05 was considered statistically significant.
4. While the study identifies key proteins and pathways affected by SPOP deficiency, it lacks in-depth mechanistic exploration of how these changes lead to the observed phenotypes. Enzymes for lipid synthesis and metabolism should be examined.

Response: We appreciated the reviewer’s suggestion. In the current study we profiled the protein expression in the liver by mass spectrometry in the *Spop* CKO mice, KEGG pathway analysis revealed that downregulated proteins were mostly enriched in fatty acid biosynthesis (Fig 3h). Notably, we found several known metabolic proteins that had critical impacts on fatty acid synthesis, including malonyl-CoA-acyl carrier protein transacylase (MCAT) which catalyzed the transfer of a malonyl group from malonyl-CoA to the mitochondrial acyl carrier protein, acetyl-CoA carboxylase beta (ACACB), and acetyl-CoA carboxylase alpha (ACACA) which functioned as the rate-limiting step in fatty acid synthesis, to be significantly downregulated in the *Spop* CKO mice.

To further explore the potential mechanism underlying the phenotypes of the *Spop* CKO mice, We also quantified mRNA levels of two critical genes involved in fatty acid metabolism in the BAT, our results indicated that the expression of the transcription factor Pgc1 which was associated with lipid oxidation was upregulated in *Spop* CKO mice, while the expression of fatty acid synthase (FASN) which was a vital enzyme in fatty acid synthesis was downregulated (Fig S4).

5. The manuscript discusses the findings in the context of obesity and metabolic diseases but does not sufficiently address the translational potential of these findings.

Response: we have added content on the translational potential of targeting SPOP for the treatment of obesity in the discussion section.

SPOP has been a frequently mutated hotspot, especially in many cancer types. Mutations that affect evolutionarily conserved residues in the substrate-binding MATH domain are predicted to result in the dysfunction of SPOP. Guo et al. report the development of a specific SPOP inhibitor that would efficiently inhibit the ubiquitination and degradation of tumor repressors in kidneys (Zhong-Qiang Guo). It would be reasonable to speculate that the “loss of function” mutations of and inhibition of SPOP might lead to reduced adipogenesis based on the current study. However, further insights into the mechanistic basis of SPOP mediated regulation of adipogenesis and thermogenesis are still needed to explore the therapeutic potential of targeting SPOP.
6. Some figures lack clarity and detailed explanations in the text.

Response: We thank the referee for this suggestion. We have added more descriptions to corresponding figures, for example, more descriptions in Fig3, also we have corrected a mistake in Fig4.
7. There are grammatical errors and awkward phrasings throughout the manuscript.

Response: We followed the reviewer’s suggestion. The whole paper has been re-edited by a professional native English speaker.
8. There is some redundancy in the introduction and discussion sections, where similar points are reiterated. Streamlining these sections to avoid repetition would make the manuscript more concise and focused.

Response: Thanks for your suggestion. We found the redundancy and had modified the corresponding contents in the discussion.

Reviewer #2: This manuscript reports altered adipogenesis and thermogenesis in mice lacking SPOP in Fstl1-expressing cells. While the phenotype is interesting, some limitations in the experimental design were noticed. Also, details of some experiments that are required to interpret the data are missing.
1. More details about the Cre line used in this study are needed. What cell types express Fstl1-CreERT2?

Response: *Fstl1-Cre^ERT2^* knock-in mice were used in this study, and the CRE gene was whole-body expressed. Therefore, the knockout of SPOP was not tissue or cell-type-specific because CRE was expressed in every kind of cell type. Knockout of SPOP happened only after the injection of Tamoxifen in mice. We have made this clear in the materials and methods.
2. The study is about adipogenesis and thermogenesis. Why did the authors choose the kidney for the proteomics analysis and KO validation western blot in Figure 1? Changes in the kidney do indicate what happens in other tissues.

Response: We thank the referee for pointing out this. As a substrate adaptor of cullin 3-based E3 ligase, SPOP is involved in tumor development and progression by promoting ubiquitination and degradation of its substrates in human cancers, typically in kidney cancer. The cytoplasmic SPOP serves as a regulatory hub in order to promote kidney cancer through the ubiquitination and degradation of multiple cancer proteins that are involved in cellular proliferation and apoptosis. SPOP plays a tumor-promoting role in kidney cancer. For example, clear cell renal cell carcinomas (ccRCCs) are the most common form of kidney cancer, accounting for 75% of all cases. SPOP protein is highly expressed in 99% of ccRCCs, the most prevalent form of kidney cancer.

In the current study we first carried out a proteomic assay of the *Spop* knock-out mice in order to explore the potential targets of SPOP. The initial aim of the assay was to identify proteins that were modulated by SPOP in mouse tissue. Considering the critical role of SPOP in the development of kidney cancer, we selected the kidney for the proteomic assay. KEGG pathway analysis showed that differentially expressed proteins were significantly enriched in metabolism pathways, including regulation of lipolysis in adipocytes, fatty acid biosynthesis, fatty acid metabolism, fatty acid degradation and glycerolipid metabolism. To our note, UCP1, which is also expressed in the renal tubular epithelial cells of normal kidneys besides in the brown adipose tissue, was significantly upregulated in the *Spop* KO mice. We also found higher expression of UCP1 in the BAT of *Spop* KO mice (Fig 4c). All of these data led us to further investigate the function of SPOP in the regulation of adipogenesis and thermogenic capacity in mice.
3. What is the genotype of WT mice used in this study?

Response: In the current study, we used a conditional knockout strategy to generate the *SPOP* knockout mice. The knockout of *SPOP* happened upon the injection of tamoxifen into mice. We used the *Spop^F/F^Cre^ERT2+/+^* mice without tamoxifen injection as the wild-type controls, and the *Spop^F/F^Cre^ERT2+/+^* mice were intraperitoneally injected with tamoxifen at the dose of 175 mg/kg bodyweight three times every other day as knockout mice. We have clarified the WT mice in the materials and methods.
4. Supplemental figure legends are missing.

Response: We thank the reviewer for pointing this out. As the reviewer suggested, we have added the Supplemental Figure legends into the manuscript.
5. Figure 3 shows a higher relative weight for all tested organs in KO vs. WT. Which organ(s) in the KO have reduced weight compared to WT to cause less body weight gain in these mice? What are the organs’ absolute weights?

Response: We appreciate this suggestion. Actually the body weight of the Spop CKO mice decreased compared with the wild-type controls as we showed in Fig 3a. The absolute weights of the liver, WAT and BAT showed significant decrease in the Spop CKO mice. We have included the organs’ absolute weights in Fig 3b. We also modified the corresponding description in the manuscript.

6. Quantifications for all western blots and histological images are needed.

Response: We thank the reviewer for pointing this out. We have quantified all the western blotting figures and incorporated the data (Fig 1b, 2f, 4d and 5b).
7. Lines 182-184. “Wild-type mice were substantially more cold-tolerant compared with the CKO mice, as their body temperature started to drop significantly after 3 hr of cold challenge (Fig 4a).” Is this what the authors are trying to describe?

Response: We thank this referee for pointing out this mistake. We had meant to say that “Wild-type mice were substantially less cold-tolerant”. We have corrected this mistake in the manuscript.
8. Figure 5. The efficiency of the SPOP knockdown needs to be validated. Also, the experiment is better done using primary cells from WT and KO mice.

Response: We followed the reviewer’s suggestion and carried out these assays in primary preadipocytes isolated from the WT and CKO mice. Meanwhile, knockdown efficiency of SPOP was validated by western blotting. The results from primary preadipocytes showed similar trends as what we observed in NIH3T3-L1 cells. We have updated the data in primary preadipocytes as Fig 5.

**Have all** **data underlying the figures and results presented in the manuscript been provided?**
Large-scale datasets should be made available via a public repository as described in the *PLOS Genetics* [data availability policy](http://c05y1x9s.r.us-east-2.awstrack.me/L0/http:%2F%2Fjournals.plos.org%2Fplosgenetics%2Fs%2Fdata-availability/2/010f0191b89a4ece-d67166e8-0228-49a2-b8d6-574471ff6b83-000000/F_wlt0tYIgdEQWgAArlLfnbmy8Q=175), and numerical data that underlies graphs or summary statistics should be provided in spreadsheet form as supporting information.

Reviewer #1: Yes

Reviewer #2: **No:** This study include RNAseq and proteomics data. However, the manuscript provides no access to the raw data.

Response: For the data underlying the figures and results presented in the manuscript, we are currently working on it to submit it to NCBI. We do not need to ask for an exemption for the data availability. There is something wrong with the data format so we have not submitted them to NCBI successfully yet. I think we will figure out this problem and get the accession numbers in ten days. We promise that all the data will be available via a public repository according to the PLOS Genetics data availability policy and add the accession numbers to the proof if this paper is accepted.

PLOS authors have the option to publish the peer review history of their article ([what does this mean?](https://c05y1x9s.r.us-east-2.awstrack.me/L0/https:%2F%2Fjournals.plos.org%2Fplosgenetics%2Fs%2Feditorial-and-peer-review-process%23loc-peer-review-history/1/010f0191b89a4ece-d67166e8-0228-49a2-b8d6-574471ff6b83-000000/jw1REkMbX0cQSVnHQAnZioReqKk=175)). If published, this will include your full peer review and any attached files.


If you choose “no”, your identity will remain anonymous but your review may still be made public.


**Do you want your identity to be public for this peer review?** For information about this choice, including consent withdrawal, please see our [Privacy Policy](https://c05y1x9s.r.us-east-2.awstrack.me/L0/https:%2F%2Fwww.plos.org%2Fprivacy-policy/1/010f0191b89a4ece-d67166e8-0228-49a2-b8d6-574471ff6b83-000000/1Z_lkN_N3cqE8Ql411v4-gB-9PU=175).

Reviewer #1: **Yes:** JUN REN

Reviewer #2: No
